# Multidimensional machine learning algorithms to learn liquid velocity inside a cylindrical bubble column reactor

**DOI:** 10.1038/s41598-020-78388-x

**Published:** 2020-12-09

**Authors:** Meisam Babanezhad, Azam Marjani, Saeed Shirazian

**Affiliations:** 1 Institute of Research and Development, Duy Tan University, Da Nang, 550000 Viet Nam; 2 Faculty of Electrical – Electronic Engineering, Duy Tan University, Da Nang, 550000 Viet Nam; 3 Department for Management of Science and Technology Development, Ton Duc Thang University, Ho Chi Minh City, Viet Nam; 4 Faculty of Applied Sciences, Ton Duc Thang University, Ho Chi Minh City, Viet Nam; 5 Laboratory of Computational Modeling of Drugs, South Ural State University, 76 Lenin prospekt, Chelyabinsk, Russia 454080

**Keywords:** Mathematics and computing, Physics

## Abstract

For understanding the complex behavior of fluids in a multiphase chemical bubble column reactor, a combination of the computational fluid dynamic (CFD) method and the adaptive network-based fuzzy inference system (ANFIS) method is used to predict bubble flow inside a reactor based on the function of column height. In this study, the Euler–Euler model is employed as a CFD method. In the Eulerian method, continuity and momentum governing equations are mathematically computed for each phase, while the equations are connected together by source terms. After calculating the flow pattern and turbulence flow in the reactor, all data sets are used to prepare a fully artificial method for further prediction. This algorithm contains different learning dimensions such as learning in different directions of reactor or large amount of input parameters and data set representing “big data”. The ANFIS method was evaluated in three steps by using one, two, and three inputs in each one to predict the liquid velocity in the x-direction (Ux). The x, y, and z coordinates of the location of the node of the liquid were considered as the inputs. Different percentages of data and various iterations and membership functions were used for training in the ANFIS method. The ANFIS method showed the best prediction using three inputs. This combination also shows the ability of computer science and computational methods in learning physical and chemical phenomena.

## Introduction

In general, the usage of bubble column reactors (BCRs) in multiphase mode for mixing solid, liquid, and gas phases can produce a variety of products in many industries. In particular, the mixing of gas and liquid creates physical and chemical reactions that are used in the wastewater, pharmaceutical, and biotechnology industries^1–5^. BCRs are more prevalent in industry owing to proper functioning, structure simplicity, fast performance, low cost during computational fluid dynamic design, and artificial intelligence design^6^. These novel types of reactors can increase gas and liquid contact area or interfacial area. This provides phase mixing and high mass transfer rates. Besides, various catalysts can be used at any time to enhance chemical reactions. The scale of bubble column reactors and their optimization depends on the complex behavior of gas flow in the liquid phase and the sparger specifications^7–13^. In addition to bubble column specifications, the size of bubbles and the interaction between bubbles affects the overall scale-up and optimization of the reactor^5,14–18^.

Simulation of bubble column reactor makes such data as pressure, temperature, velocity, and the measure of gas content. These data provide a detailed understanding of the bubble column design and the two-phase fluid flow complexity^19–25^. Although several numerical and experimental methods estimate the multiphase flow pattern in BCRs^1–3,13,24–27^, there are still some obstacles to simulate the gas flow through a constant liquid fully. Probe sensors also disrupt the bubbles inside the bubble column reactor by creating vortex. Measuring fluid flow by microscopic and high-speed cameras is expensive and is also limited to substantial frames. Computational time is another limitation to predict large BCRs at different times and operating conditions^6^. According to the above limitations, artificial intelligence algorithms generally show the ability to cooperate with the experimental and CFD methods by accurately understanding fluid flow and the reduction of computational time^20,28–32^. Soft computing methods such as neural networks^33–35^, support vector machines, evolutionary algorithms, simulated annealing, and the adaptive neuro-fuzzy inference system (ANFIS) have been suggested in the literature for simulation of real-life applications^36–39^. Moreover, it is worth mentioning that ANFIS can be used as an assistant tool besides CFD. The high capability of ANFIS for training the CFD data helps not to rerun the CFD simulations, and it can find the middle points and optimization can speed up. Also, ANFIS has high capability in the training of the large datasets and big data; it can find data in local nodes. Furthermore, each of x, y, and z computing nodes in the BCR can predict characteristics of the fluid and turbulence properties, and it can create an artificial BCR. Moreover, the method fully depends on the domain of the particular data that is trained in CFD. Also, the ANFIS can have the capability based on the trained inputs, and it can find the effect of the inputs on the output. This method can find the effective parameter in process engineering; however, one cannot find this effect when the number of inputs increases.

ANFIS method with the ability to learn many physical models can be advantageous in this context, and main processes of chemical and biochemical engineering^40–42^. The presence of humans in places where chemical reactions happen is hazardous regarding health. The use of robots instead of humans is expanding. The ANFIS method is also developing to predict complex engineering mechanisms for controlling the movement of robots^40^. The ANFIS method has complex algorithms and can provide a very smart way to make decisions and correct its accuracy when it comes to very difficult choices^43–47^. There are several methods for teaching ANFIS algorithms in literature^6,48^. The learning in this method depends entirely on the simulation data or empirical output^40^. Recently, an ANFIS method was used to simulate the flow pattern in a BCR. They mainly used a new combination of soft-computing with the CFD method that was suggested in Pourtousi’s researches^49–51^ to generate a new domain of data through big data. They used the multiphase reactor hydrodynamics information for the training phase. They found that the CFD method plus the ANFIS method is a significant overview to simulate BCRs properties^9,26,52^.

The method of ANFIS was frequency used for the prediction of gas hold-up and turbulence characteristics, such as turbulence kinetic energy in the bubble column reactor^50,53,54^. This method was examined in prior research to explore the gas distribution in the reactor for different input parameters, such as x, y, and z computing nodes and sparger specifications (such as ring sparger diameter). However, in previous work, each input parameter was not examined individually with flow distribution with regards to model’s accuracy and prediction capability.

It was reported that the ANFIS method could be used for the simulation of the bubble flow in the BCR instead of CFD methods when the flow regime is uniform, and most of the bubbles have the same velocity and spherical shape^55,56^. In this study, the ANFIS simulation was tested in three steps by using one, two, and three inputs in each one to predict the liquid velocity in the x-direction (Ux). The x, y, and z coordinates of the liquid were considered as the inputs. The ability of the ANFIS method is examined by different types of data from low to extensive.

The computational fluid dynamics is used to calculate liquid flow distribution in the reactor. For this purpose, the Euler–Euler CFD method is used to simulate gas–liquid interaction in the reactor and compute liquid flow pattern and gas hold-up in the bubble column reactor. As the Euler–Euler CFD method, which is a numerical method, provides data in several local nodes in the reactor. The data are selected for the training of AI method. The AI method can understand the process at local nodes and provide a mapping framework for the prediction of the flow in the whole of the column. In this study, this intelligent algorithm's strength is confirmed by comparing the outputs of the ANFIS model with those of Euler–Euler (E–E) one.

## Methodology

### Geometrical structure

In this research, an industrial BCR was used at room temperature of 23 °C and atmospheric pressure. At the end of this reactor, the ring sparger has 20 orifices with a diameter of 0.7 mm. The ring of sparger orifices is arranged neatly at regular intervals at the end of the bubble column reactor. The shape of bubbles is supposed to be spherical. Besides, the least collision and the smallest break up are considered to happen for the bubbles. The orifices also generate a superficial gas velocity of 0.005 m/s at the bubble column reactor (BCR).

Moreover, the height of the 3D cylindrical BCR is 2.6 m, and its diameter is 0.288 m, and it is filled with stationary water. Twenty similar holes are at the bottom of the BCR with a diameter of 0.007 m. The superficial gas velocity for the simulation cases is 0.005 m/s, leading to a homogenous regime in which the bubbles sizes, velocities, and shapes are the same.

### CFD modeling

For the CFD simulation of the study, ANSYS CFX software is used. Moreover, the ANIFS method is run in the MATLAB framework. Also, the Euler–Euler method uses a general understanding of the gas movement inside the bubble column reactor. In this method, continuity and momentum transfer equations^6,20,27^ are defined as follows:

1 $$\frac{\partial }{\partial \mathrm{t}}\left({\uprho }_{\mathrm{k}}{\upepsilon }_{\mathrm{k}}\right)+\nabla \left({\uprho }_{\mathrm{k}}{\upepsilon }_{\mathrm{k}}{\mathbf{u}}_{\mathrm{k}}\right)= 0$$

2 $$\frac{\partial }{\partial \mathrm{t}}\left({\uprho }_{\mathrm{k}}{\upepsilon }_{\mathrm{k}}{\mathbf{u}}_{\mathrm{k}}\right)+\nabla \left({\uprho }_{\mathrm{k}}{\upepsilon }_{\mathrm{k}}{\mathbf{u}}_{\mathrm{k}}{\mathbf{u}}_{\mathrm{k}}\right)=-\nabla \left({\upepsilon }_{\mathrm{k}}{\uptau }_{\mathrm{k}}\right)-{\upepsilon }_{\mathrm{k}}\nabla \mathrm{P}+{\upepsilon }_{\mathrm{k}}{\uprho }_{\mathrm{k}}\mathrm{g}+{\mathbf{M}}_{\mathrm{I},\mathrm{k}}$$

where *ε*_*k*_ expresses the fraction, and *u*_*k*_ represents the average velocity of the discrete and continuous phases^7^.

The finite volume method is used for the discretization of computational non-structure nodes. This generation of nodes enables us for easy implementation and generation of nodes in the domain. After the discretization of each node, gas bubble characteristics are resolved, and then they are coupled with matrix phase calculation. For the movement of gas bubbles, the constant drag force is used throughout the domain, which represents spherical bubble dynamics in the experimental study.

The momentum transfer equation describes stress, gradient pressure, gravity, and the exchange of the range of motion between a dispersed and continuous phase during phase interaction. The term stress for a dispersed phase can be described as follows^6^:

3 $${\uptau }_{\mathrm{k}}=-{\upmu }_{\mathrm{eff},\mathrm{k}}\left(\nabla {\mathbf{u}}_{\mathrm{k}}+{\left(\nabla {\mathbf{u}}_{\mathrm{k}}\right)}^{\mathrm{T}}-\frac{2}{3}\mathrm{I}\left(\nabla {\mathbf{u}}_{\mathrm{k}}\right)\right)$$

4 $${\upmu }_{\mathrm{eff},\mathrm{L}}={\upmu }_{\mathrm{L}}+{\upmu }_{\mathrm{T},\mathrm{L}}+{\upmu }_{\mathrm{BI},\mathrm{L}}$$

where *μ*_*eff, L*_ represents the effective viscosity as a function of the molecular viscosity *μ*_*L*_, the turbulent viscosity *μ*_*T,L*_ and turbulence viscosity induced by the bubbles motion *μ*_*BI,L*_.

The effective dispersive phase viscosity *μ*_*eff,G*_ based on the effective viscosity *μ*_*eff,L*_ can be written as follows:

5 $${\upmu }_{\mathrm{eff},\mathrm{G}}=\frac{{\uprho }_{\mathrm{G}}}{{\uprho }_{\mathrm{L}}}{\upmu }_{\mathrm{eff},\mathrm{L}}$$

Sato and Sekoguchi model is applied in the calculation of the two-phase flow. It is used for the purpose of simulation of the turbulence by the movement and interactions of bubbles.

The total interfacial force for the interaction between a dispersed and continuous phase can be defined as follows^51^:

6 $${\mathbf{M}}_{\mathrm{I},\mathrm{L}}=-{\mathbf{M}}_{\mathrm{I},\mathrm{G}}={\mathbf{M}}_{\mathrm{D},\mathrm{L}}+{\mathbf{M}}_{\mathrm{TD},\mathrm{L}}$$

Since bubbles do not collide and do not stick together, uniform shapes are created. Therefore, the simplest drag model with the uniform spherical bubble shape is considered. The drag model coefficient can represent the motion of a spherical bubble. The drag model is the main force of modeling the bubble column reactor, especially when the bubble column reactor is homogeneous^7,57^.

The following formula shows the calculation of drag force ***M***_*D,L*_, where *C*_*D*_ is the drag coefficient, and *d*_*B*_ is the diameter of the bubble^51^.

7 $${\mathbf{M}}_{\mathrm{D},\mathrm{L}}=-\frac{3}{4}{\in }_{\mathrm{G}}{\uprho }_{\mathrm{L}}\frac{{\mathrm{C}}_{\mathrm{D}}}{{\mathrm{d}}_{\mathrm{B}}}\left|{\mathbf{u}}_{\mathrm{G}}-{\mathbf{u}}_{\mathrm{L}}\right|\left({\mathbf{u}}_{\mathrm{G}}-{\mathbf{u}}_{\mathrm{L}}\right)$$

The turbulent dispersion force between a scattered and continuous phase can be calculated from the following formula^58^:

8 $${\mathbf{M}}_{\mathrm{TD},\mathrm{L}}=-{\mathbf{M}}_{\mathrm{TD},\mathrm{G}}=-{\mathrm{C}}_{\mathrm{TD}}{\uprho }_{\mathrm{L}}\mathrm{k}\nabla {\in }_{\mathrm{L}}$$

An acceptable selection of the turbulence model is necessary for predicting the hydrodynamics of the BCR. A zero-equation turbulence model is applied for the disperse bubbly phase; nevertheless, the standard *k–ε* model is used for the continuous phase. Using the *k–ε* model is advantageous because of various reasons. First, it includes low computational times. Second, it is simple, and third, it can obtain average results.

In the above formula, *k* represents liquid turbulent kinetic energy (TKE), and *C*_*TD*_ represents the turbulent dispersion coefficient. Our use of turbulent modeling to better observe gas motion, which is turbulent behavior, results in accurate modeling of bubble flow^13^.

9 $${\upmu }_{\mathrm{T},\mathrm{L}}={\uprho }_{\mathrm{L}}{\mathrm{C}}_{\upmu }\frac{{\mathrm{k}}^{2}}{\upvarepsilon }$$

The kinetic turbulent (*k*) energy equation is defined as follows:

10 $$\frac{\partial }{\partial \mathrm{t}}\left({\uprho }_{\mathrm{L}}{\in }_{\mathrm{L}}\mathrm{k}\right)+\nabla \left({\uprho }_{\mathrm{L}}{\in }_{\mathrm{L}}{\mathbf{u}}_{\mathrm{L}}\mathrm{k}\right)=-\nabla \left({\in }_{\mathrm{L}}\frac{{\upmu }_{\mathrm{eff},\mathrm{L}}}{{\upsigma }_{\mathrm{k}}}\nabla \mathrm{k}\right)+{\in }_{\mathrm{L}}\left(\mathrm{G}-{\uprho }_{\mathrm{L}}\upvarepsilon \right)$$

The energy dissipation rate (*ε*) is defined as follows:

11 $$\frac{\partial }{\partial \mathrm{t}}\left({\uprho }_{\mathrm{L}}{\in }_{\mathrm{L}}\upvarepsilon \right)+\nabla \left({\uprho }_{\mathrm{L}}{\in }_{\mathrm{L}}{\mathbf{u}}_{\mathrm{L}}\upvarepsilon \right)=-\nabla \left({\in }_{\mathrm{L}}\frac{{\upmu }_{\mathrm{L},\mathrm{eff}}}{{\upsigma }_{\upvarepsilon }}\nabla\upvarepsilon \right)+{\in }_{\mathrm{L}}\frac{\upvarepsilon }{\mathrm{k}}\left({\mathrm{C}}_{\upvarepsilon 1}\mathrm{G}-{\mathrm{C}}_{\upvarepsilon 2}{\uprho }_{\mathrm{L}}\upvarepsilon \right)$$

Various constant values are selected in the k–ε turbulence model for calculation of turbulence behavior of liquid (continuous phase) in the bubble column reactor. These values are defined as:

$$C\mu =0.09$$, $${\sigma }_{k}=1$$, $${\sigma }_{\varepsilon }=1$$, $${C}_{\varepsilon 1}=1.44$$, and $${C}_{\varepsilon 2}=1.92$$.

#### Grid

In this study, grids were used to create a three-dimensional gas–liquid motion similar to Laborde-Boutet et al.^28^ as a non-uniform grid method, and a cylindrical amplitude was used to calculate the E–E method. The reactor section is also classified non-uniformly to 60 levels and is meshed to calculate the E–E method. Unstructured meshes are used in the study, and they are non-uniform hexahedral grid meshes, which are repeated in each cross-section. This type of element is used through the reactor's domain, and in different levels of the reactor, a similar mesh pattern is designed. A non-uniform pattern is used through the domain and the specification of meshes for skewness ≈ 0.6, aspect ratio ≈ 3, and orthogonal quality ≈ 0.6. For the best quality of mesh, mesh sensitivity assessment is considered in this study. The number of elements more than 40,000 grids is in good agreement with existing numerical and experimental results in the literature.

#### Specifications of the boundary conditions and interfacial force models

A ring sparger is applied in BCR, and for modeling it, the source point at the end of the BCR is used. On the top of the BCR, a degassing boundary condition is used for modeling the gas outlet. For the gas phase, a free slip boundary condition is applied. Furthermore, on solid walls, a no-slip boundary condition is utilized for the liquid. The drag coefficient is applied as the drag model, which helps to model spherical bubble movements with uniform shapes, and without any interactions inside the BCR, including coalescence and breakup. Therefore, no interaction is considered for the bubbles in the BCR. Also, the drag coefficient is equal to 0.44. The bubble diameter is 4 mm that is based on the industrial BCRs suggested by Pfleger and becker’s research^59^. A lift model for modeling the bubble movements is not needed due to the homogeneity of the flow. The drag model can be 100 times more dominant rather than other interfacial force scheme models. The CFD results are time-averaged for 1400 s CFD simulation time. The time-averaged calculations are applied for turbulent properties and gas–liquid flow patterns inside the BCR. The sensitivity of time steps is studied among values, which are ranged 0.1–0.01. The time step is 0.1 that is a suitable value in order to track both gas and liquid interaction in the BCR with Courant–Friedrichs–Levy (CFL) number less than one.

**Figure 1:**
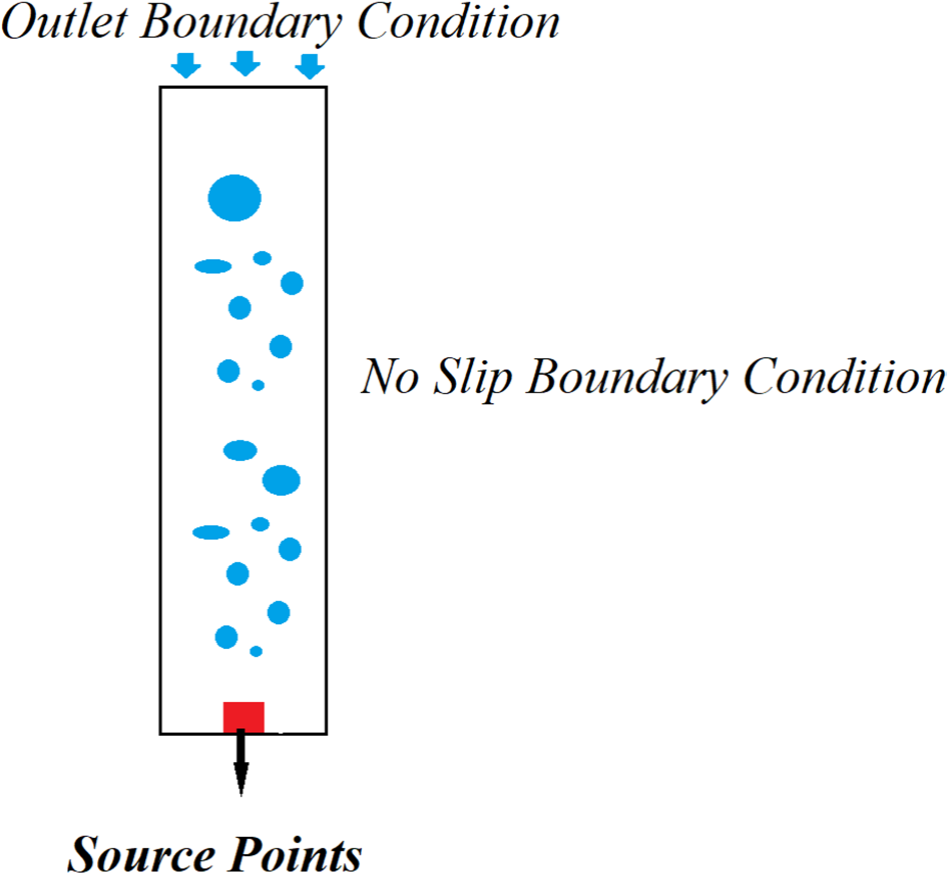
Schematic picture of the bubble column reactor with numerical boundary conditions.

The schematic shape of the bubble column reactor is shown in Fig. 1. As the figure shows, the inlet boundary conditions or ring sparger properties, such as sparger holes and superficial gas velocity, are defined as twenty source points at the reactor's bottom. However, to determine gas outlet, the degassing boundary condition is used at the reactor's top surface. Near solid walls, a no-slip boundary condition is used for the liquid/continuous phase. As a result of the reactor's homogeneous flow regime, bubbles are spherical formed through the bubble column reactor's bulk region with low bubble interaction, coalescence, and break-up rate.

### Adaptive-network-based fuzzy inference system (ANFIS)

**Figure 2:**
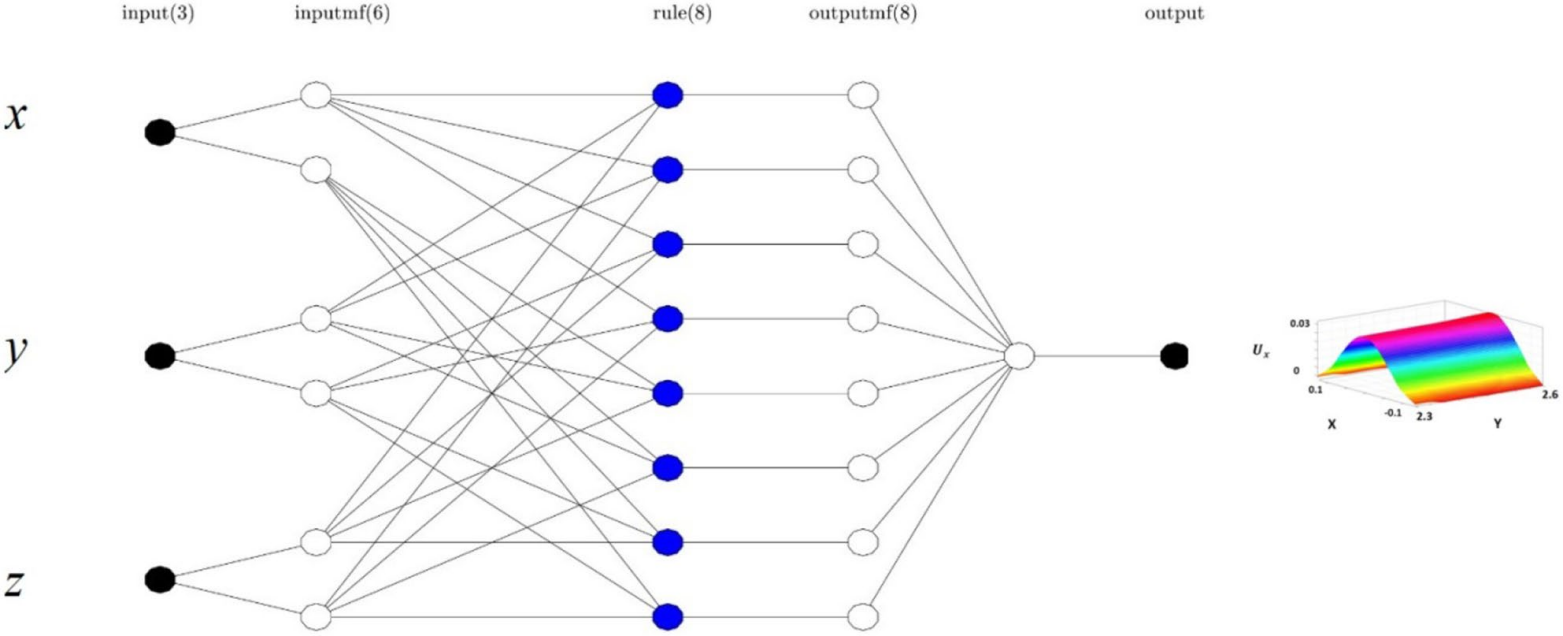
Adaptive neuro-fuzzy inference system pattern for the simulation of liquid velocity at different neural nodes. Input parameters are x, y, and z computing nodes in the bubble column reactor. Two membership functions are considered in each input parameter in the schematic figure.

To simulate the mathematical relation of complex physical and chemical behavior, a fuzzy inference structure is used, called ANFIS, and uses neural networks to learn the physical or chemical process, and fuzzy logic is used for decision making. Many studies use Takagi and Sugeno to recommend the if–then ANFIS method^60^. In the first step of the learning process, all learning data is categorized at various levels of membership formations (MFs). Membership formulations create conditions that can be fixed with the physical process to create the best description of that physical process. According to Fig. 2, the first feedback from the learning step is modified based on the AND rule. The function ith rule is expressed as:

12 $${w}_{i}={\mu }_{Ai}\left(X\right) {\mu }_{Bi}\left(Y\right){\mu }_{ci}(Z)$$

In the above function, *w*_*i*_ expresses the feedback outcoming from learning *μ*_*Ai*_, *μ*_*Bi*_, and *μ*_*Ci*_ show the incoming from learning feedback. In the learning process in the first mode, the x coordinate of nodes locations is considered as one input. The output is the fluid velocity in the x-direction (U_x_). In the second step, learning is done by two inputs in the x and y coordinate of nodes locations. In the third mode, the learning is done by three inputs of x, y, and z coordinates of nodes locations^6^.

In training data, a three-dimensional CFD mesh is captured radially and introduced into a new matrix. By using this new matrix, we can simulate different bubble column positions in the prediction process. These new bubble columns can generate data to be employed for training and learning in ANFIS. Once the model has been learnt, it can re-invoke the prediction process to predict new data in a bubble column. First, the learning step is performed with 40% data, and learning is compared with the CFD data, and the test data, which is 60% of the total amount of data not used in the learning process, is added to 40% of the training data, and a total of 100% data are compared. In the end, the prediction process is the nodes that ANFIS proposes for these nodes and has not previously learned, so these nodes are called neural network nodes. At the third level of learning, the relative firing strengths of each rule are formulated which is equal to the weight fraction of each layer on the total amount of all rules' firing strengths^6,51^:

13 $$\stackrel{-}{{w}_{i}}=\frac{{w}_{i}}{\sum \left({w}_{i}\right)}$$

In this formula, $\stackrel{-}{{w}_{i}}$ normalized firing strengths are called. In the fourth level of learning implemented the if–then rule function obtained by Takagi and Sugeno^60^. The mesh formula in ANFIS can be written as follows:

14 $$\stackrel{-}{{w}_{i}}{f}_{i}=\stackrel{-}{{w}_{i}}({p}_{i}{D}_{s}+{q}_{i}x+{r}_{i}H+{S}_{i})$$

In this formula *p*_*i*_, *q*_*i*_, *r*_*i,*_ and *s*_*i*_ are the parameters of 'if–then rules' and are called consequent parameters. To get the output of the method that shows the forecast data, all input feedback is integrated from the fourth level.

#### Membership function (MF) evaluation

The root mean square error (RMSE) equation, in which *N* is the number of test levels, can be defined as:

15 $$RMSE=\sqrt{\frac{1}{N}\sum_{i=1}^{N}{\left(Actual\,Output-Estimated\,Output\right)}^{2}}$$

The correlation coefficient (CC) formula, shows the connection strength between CFD and ANFIS, in this formula *y*_*CFD(i)*_ and *y*_*CFD(m)*_ are the Euler–Euler method and *y*_*pre(i)*_ and *y*_*pre(m)*_ prediction results from the algorithm at different levels of the reactor. It is expressed in the following:

16 $$CC=\frac{\sum_{i=1}^{N}\left({y}_{\mathrm{CFD}(i)}-{y}_{\mathrm{CFD}(m)}\right)\left({y}_{\mathrm{pre}(i)}-{y}_{\mathrm{pre}(m)}\right)}{\sqrt{\sum_{i=1}^{N}{\left({y}_{\mathrm{CFD}(i)}-{y}_{\mathrm{CFD}(m)}\right)}^{2}\sum_{i=1}^{N}{\left({y}_{\mathrm{pre}(i)}-{y}_{\mathrm{pre}(m)}\right)}^{2}}}$$

The correlation coefficient R is also considered in the analysis. The expiration of the R is written such as the following:

17 $$R=\frac{\sum_{i=1}^{N}\left({x}_{\mathrm{i}}-\stackrel{-}{x}\right)\left({y}_{\mathrm{i}}-\stackrel{-}{y}\right)}{\sqrt{\sum_{i=1}^{N}{\left({x}_{\mathrm{i}}-\stackrel{-}{x}\right)}^{2}\sum_{i=1}^{N}{\left({y}_{\mathrm{i}}-\stackrel{-}{y}\right)}^{2}}}$$

**Figure 3:**
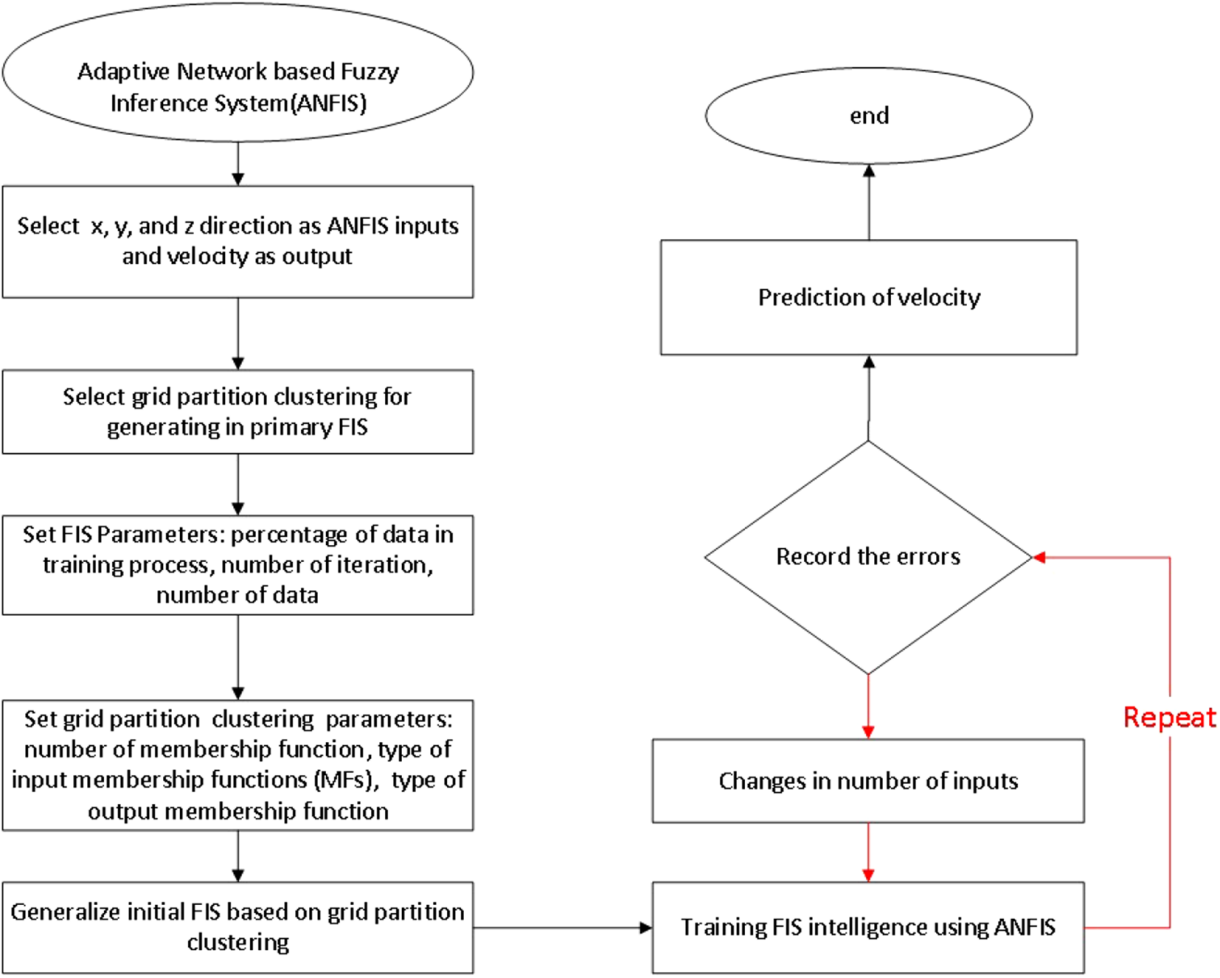
Flow chart for the model of ANFIS in predicting fluid characteristics in the bubble column reactor.

**Figure 4:**
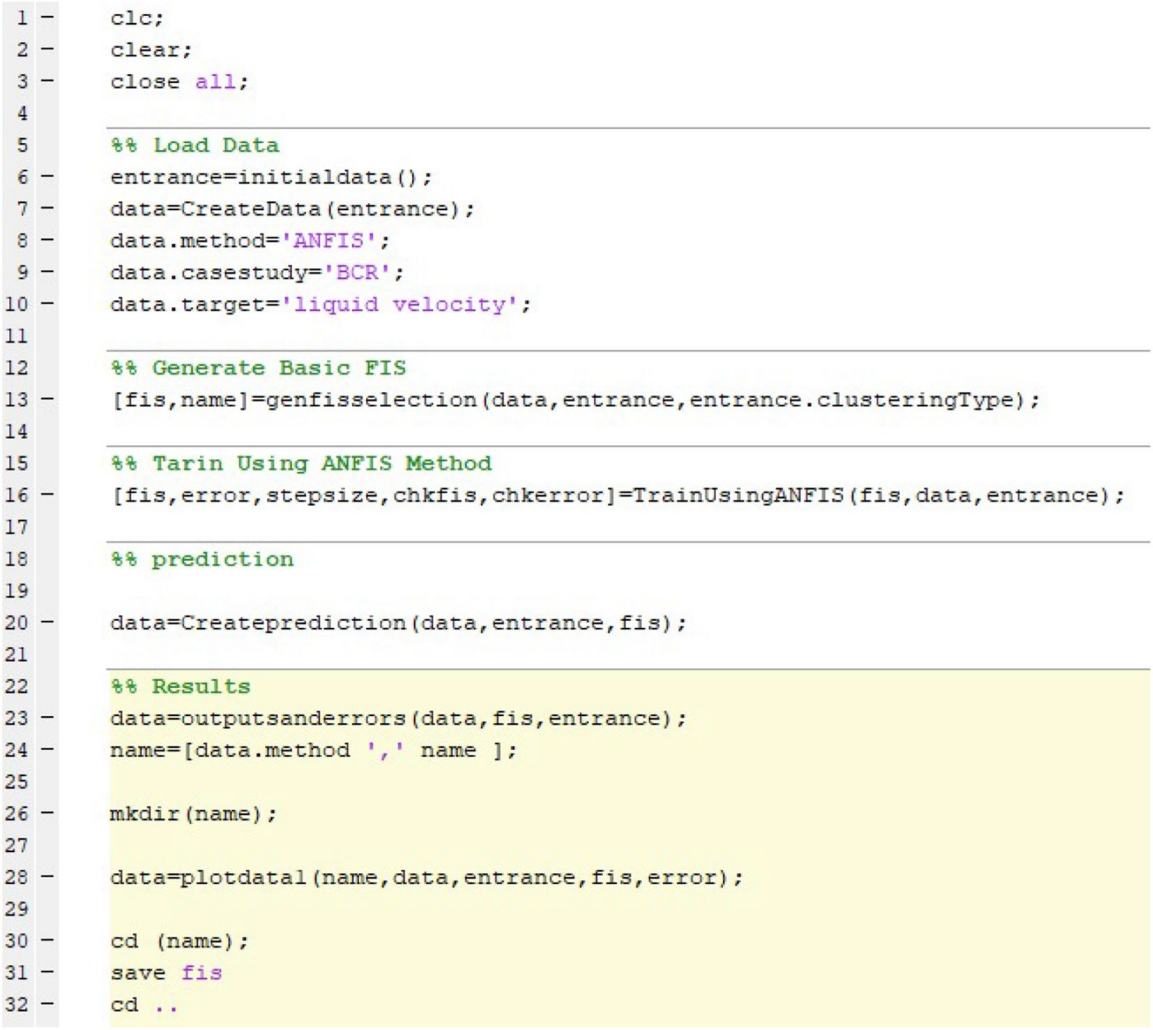
Sample MATLAB script in the training and prediction process.

To better understand the model implementation, the model of ANFIS is implemented in the MATLAB software. To run the algorithm's main core first inputs and outputs are defined in the form of a matrix in the MATLAB. In this prediction study, x, y, and z computing points, corresponding to the physical geometry specification are used as input parameters of training, while the liquid velocity distribution in the domain is defined as an output parameter in the data-driven model. To design and generate the primary FIS structure the grid partition clustering is used in the algorithm as a flexible model to specify membership specifications. In the next phase of the model parameters description, several tuning parameters are selected, such as percentage of training data, number of iteration or epoch number, and number of data for the training process. In addition to that, membership properties are defined in the model. In this section of the model, the number and type of membership functions, and the type of output membership functions are selected. After this stage, the initial FIS is defined in the model based on grid partition clustering. After all definitions of model parameters, the ANFIS method trains the FIS structure. However, the model is examined in several iterations to reach a high accuracy or a low number of errors. To improve the level of accuracy the number of inputs is changed in the model. Then, in the prediction process, the model predicts velocity distribution in the bubble column reactor (Fig. 3). The details of the MATLAB script is shown in Fig. 4. The data selection and definition of model parameters are illustrated. At the end of the scrip, the prediction part is activated.

## Results and discussion

In this study, the locations of nodes in the x, y, and z directions are selected as input variables, and the liquid velocity was selected as the critical output variable. The liquid velocity is a crucial parameter in the bubble column reactor in detecting the hydrodynamic bubble column reactor, and this parameter can significantly affect the design and scale-up of the bubble column reactor. Liquid velocity is also one of the most important characteristics in determining the flow pattern in the bubble column reactor, and this parameter indicates whether the flow is homogeneous or heterogeneous.

**Figure 5:**
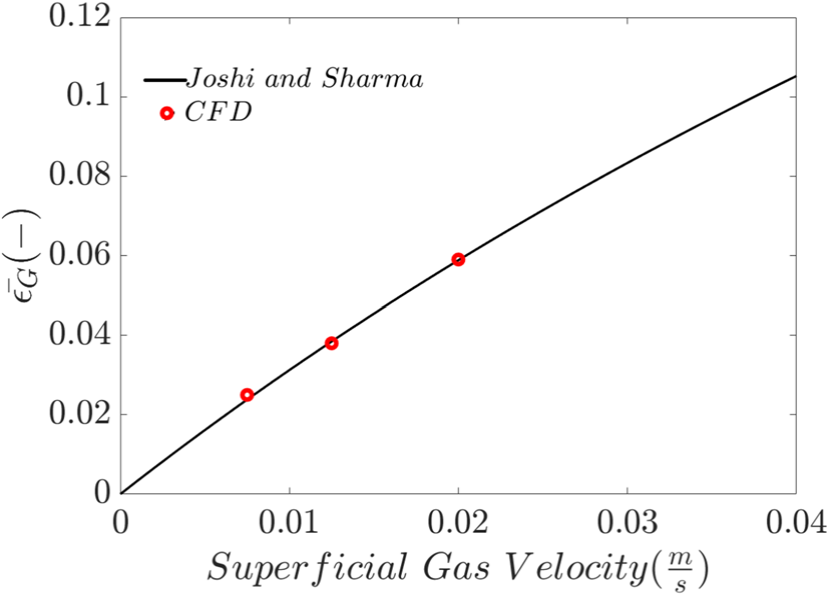
validation of current numerical method (single size Eulerian method) with existing mathematical correlations ($\frac{\mathrm{superficial \,gas \,velocity}}{0.3+2(\mathrm{superficial \,gas \,velocity})}$ ), Joshi & Sharma^61^.

Figure 5 shows the validation of the current numerical method with an existing mathematical correlation^61^. The amount of average gas fraction (gas hold-up) in the bubble column reactor, at low superficial gas velocity has a linear behavior and correlation with superficial gas velocity due to existing spherical bubbles with less turbulence interaction and coalescence or break-up phenomena. After validation of current numerical results, all datasets are trained in the machine learning method.

In the present study, there are 40,000 data referring to the entire bubble column reactor. 10% of the total data (i.e., 4000 data) corresponding to 10% of the upper bubble column reactor are selected to use in the ANFIS. After the simulation, the CFD results of 60% of local nodes participate in the training process. The remaining data are used for testing and evaluation of the predicted results. During the prediction process, the gradient descent method is used for better prediction of the data. The validation process is done by a comparison between the local CFD results and the AI results. Different evaluation criteria such as RMSE, are used for the validation process. In the learning process, 40% and 60% of the data are used for training and testing, respectively. At first, training and then after testing is done. Finally, the prediction is performed based on artificial intelligence nodes.

**Figure 6:**
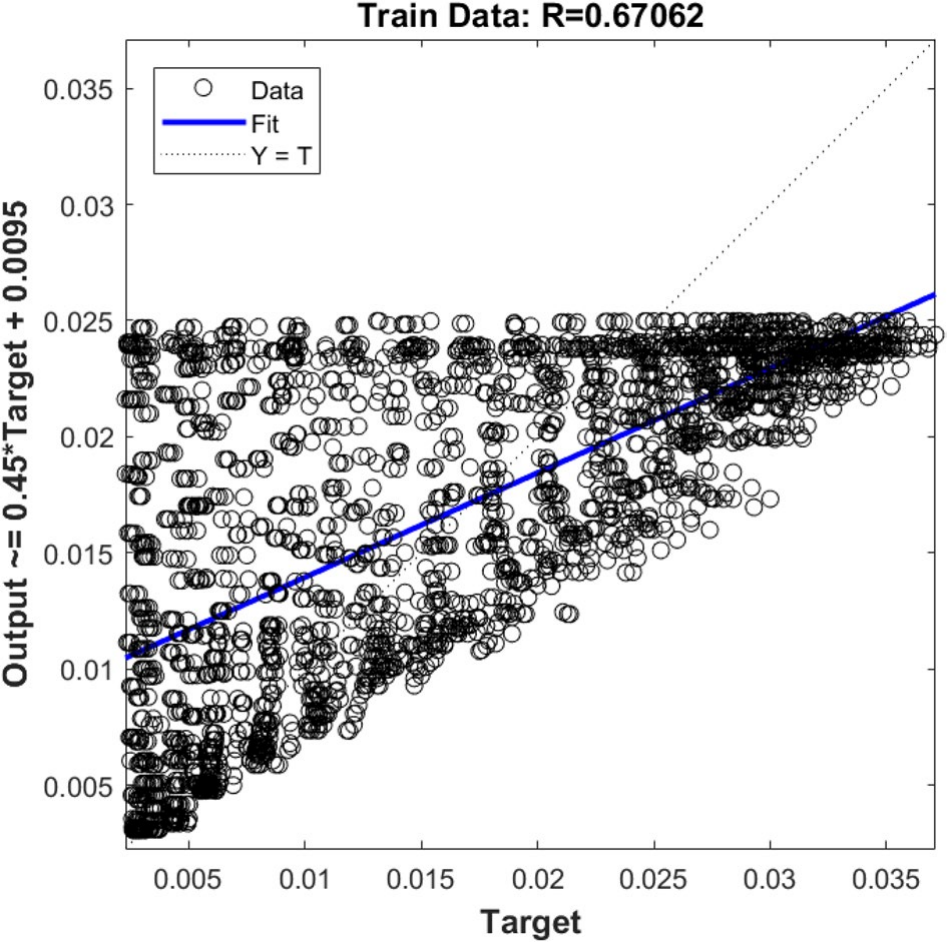
Training validation of liquid velocity for the ANFIS model with one input. The ANFIS model's output is the liquid velocity distribution, and it is validated against the velocity component (x-direction) in the CFD calculation (target value).

**Figure 7:**
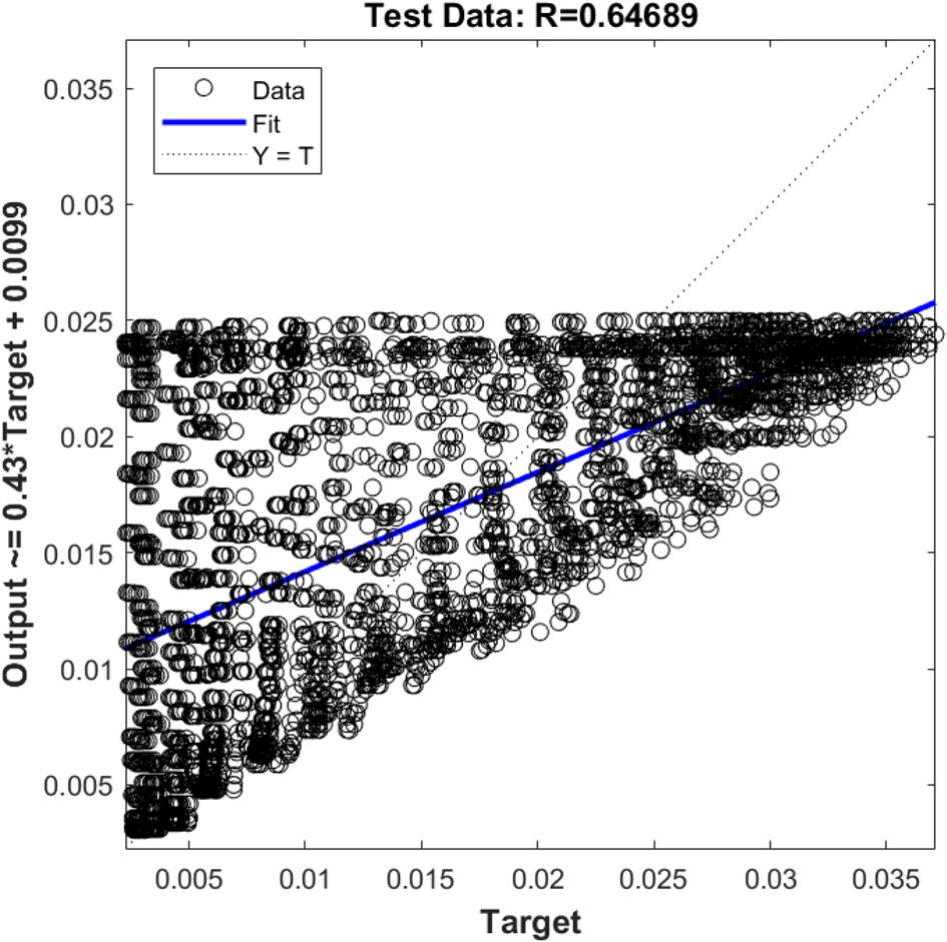
Testing validation of liquid velocity for the ANFIS model with one input. The ANFIS model's output is the liquid velocity distribution, and it is validated against the velocity component (x-direction) in the CFD calculation (target value).

In the first step, the ANFIS prediction process is done by one input (i.e., x locations of nodes), and the output is the liquid velocity in the x-direction (U_x_). Figures 6 and 7 show R values for the training and testing process, respectively. According to the Figs. 6 and 7, the method does not show a high ability. The R^2^ value, in this case, is 65%, which indicates that there is not a good agreement between ANFIS and CFD results. It seems that if the number of the rules changes or another membership formulation is used, or the amount of the available data in the training period increases, the accuracy of the method increases. However, rising the rules and increasing the learning data for the ANFIS method will increase the computational time.

**Figure 8:**
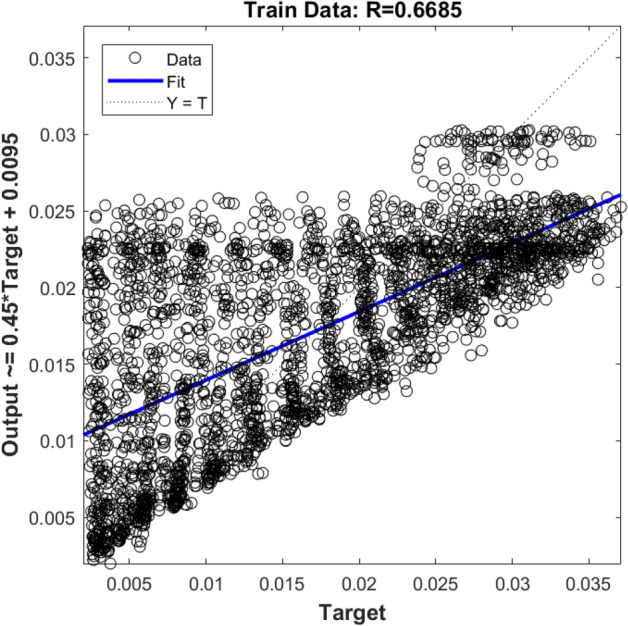
Training validation of liquid velocity for the ANFIS model with two inputs (x and Y computing nodes, the position of numerical elements). The ANFIS model's output is the liquid velocity distribution, and it is validated against the velocity component (x-direction) in the CFD calculation (target value).

**Figure 9:**
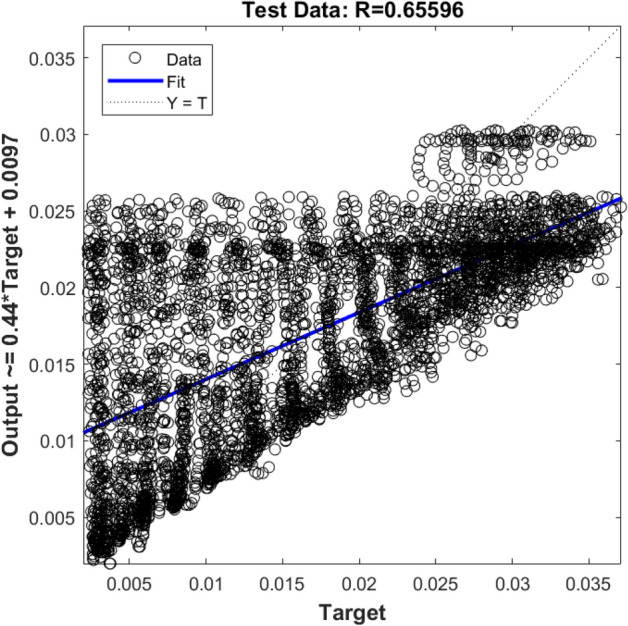
Testing validation of fluid velocity for ANFIS model with two inputs (x and Y computing node, the position of numerical elements). The ANFIS model's output is the liquid velocity distribution, and it is validated against the velocity component (x-direction) in the CFD calculation (target value).

Since one input learning was not good enough, in the next step, two inputs are used (i.e., x and y locations of nodes). As shown in Figs. 8 and 9, increasing the number of inputs, the ANFIS method still does not show an accurate prediction.

**Figure 10:**
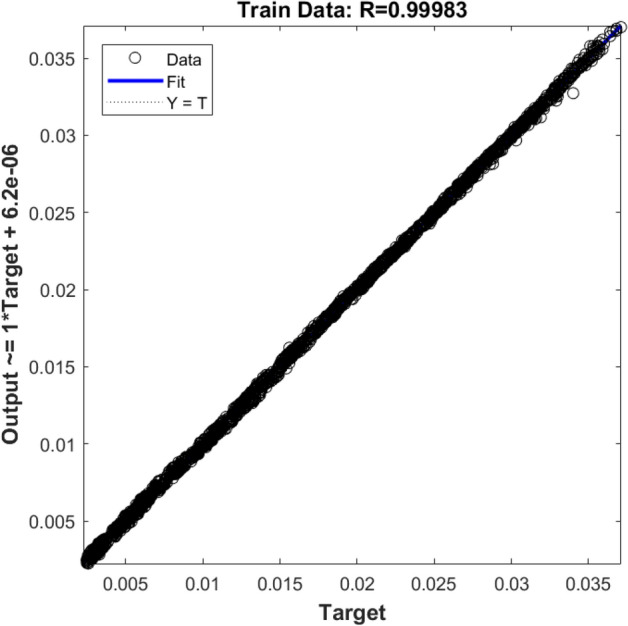
Training validation of fluid velocity for ANFIS model with three inputs (X, Y, and Z computing nodes, the position of numerical elements). The ANFIS model's output is the liquid velocity distribution, and it is validated against the velocity component (x-direction) in the CFD calculation (target value).

**Figure 11:**
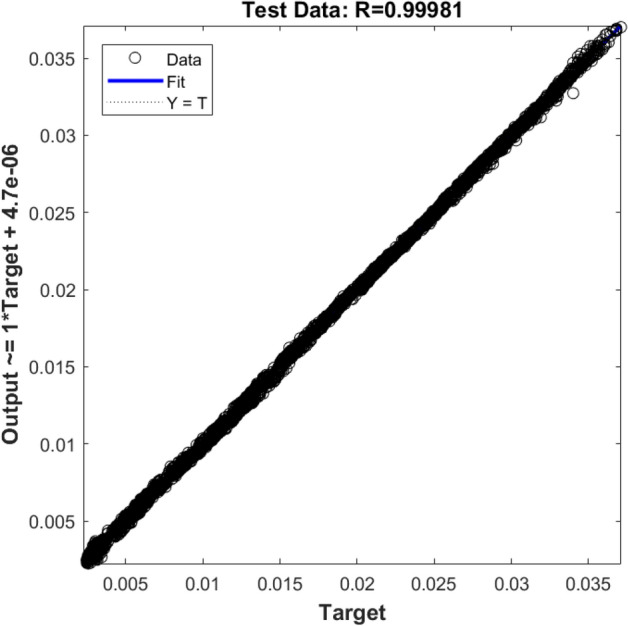
Testing the validation of liquid velocity for the ANFIS model with three inputs (X, Y, and Z computing nodes, the position of numerical elements). The ANFIS model's output is the liquid velocity distribution, and it is validated against the velocity component (x-direction) in the CFD calculation (target value).

Finally, the ANFIS method with three inputs (i.e., x, y, and z locations of nodes) is considered. For evaluating the training and testing processes for new conditions, the ANFIS method suddenly reaches the most accurate prediction. The R^2^ for training (Fig. 10) and testing (Fig. 11) exceeds 99%. This suggests the ANFIS algorithms can be a tremendous smart tool to predict fluid flow characteristics in the BCR.

As the model of ANFIS is a data-driven base framework, this model can predict the flow distribution in the bubble column reactor in the range of training datasets (input parameters). More neural cells are generated in the main core of model training by considering a high number of input parameters up to three input parameters. Therefore, the model can easily connect the input and output dataset and predict the liquid flow distribution with a high model’s accuracy (R = 0.99). The generation of a model with a low number of input parameters (input = 1) can make the model unstable in prediction capability. In this case, the model cannot find the connection between input and output parameters, and the effective input parameters can be hidden during analysis.

**Figure 12:**
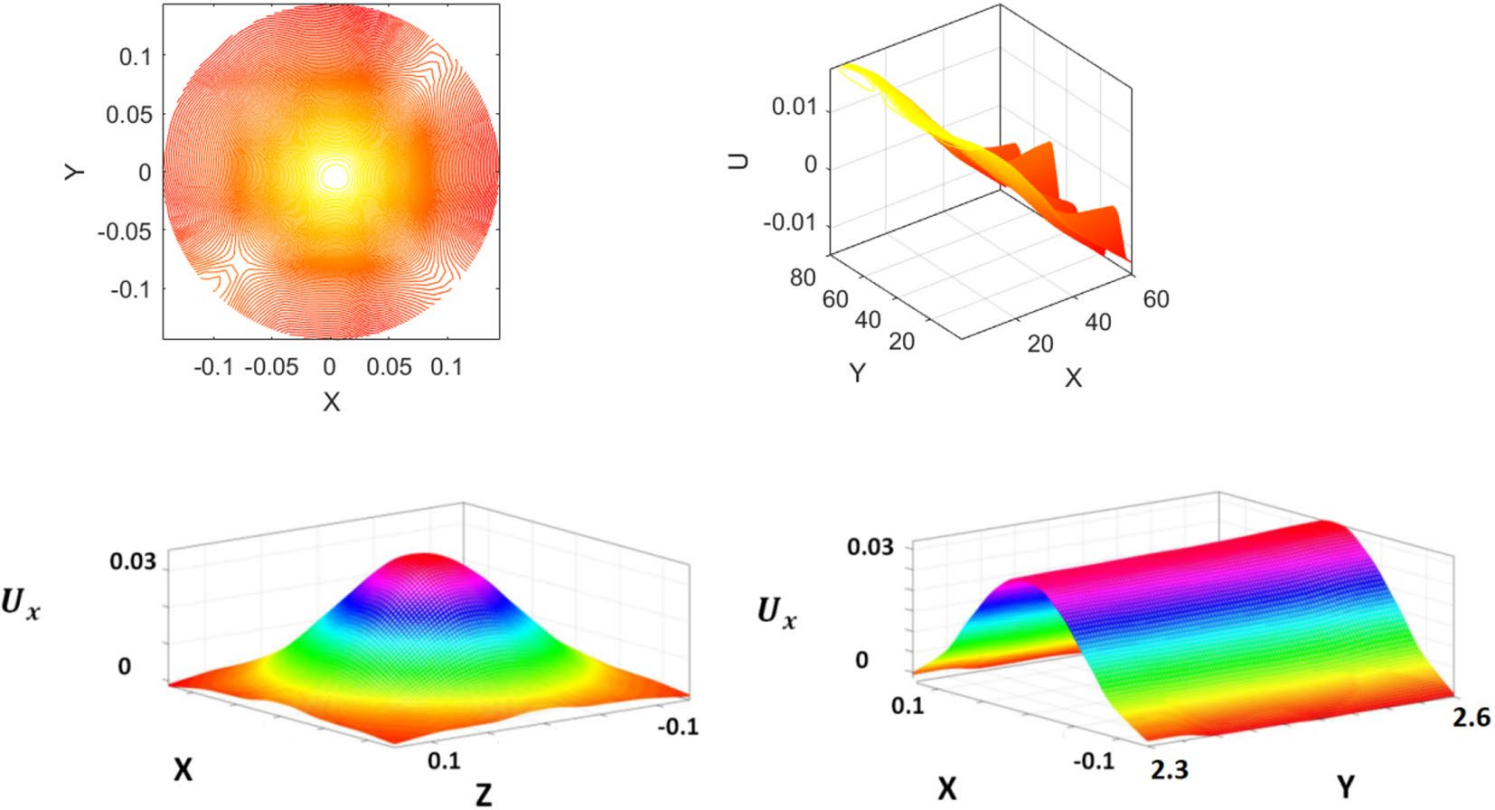
Prediction of liquid velocity for ANFIS model without using the CFD model, machine learning overview. X, Y, and Z represent computing nodes or position of numerical elements in the bubble column reactor.

At this point, the ANFIS method has reached a high level of learning ability. It means that the ANFIS method can propose all nodes itself and perform the prediction based on the proposed nodes. In this case, no CFD data is used for prediction, and this is a refinement process^40^. For example, the results of Fig. 12 have been predicted in this way. At first, the CFD results of some initial nodes were received and learned by the ANFIS method for U_x_ calculation. Then 10,000 nodes were selected for the U_x_ prediction without using the CFD data.

According to Fig. 12, the velocity in the middle of the BCR is significantly increased. This is a good ability in the BCR because when the bubbles rise up, the liquid velocity increases in the middle of the BCR, while the velocity on the side of BCR is very low. The liquid flow pattern in the column is based on maximum velocity in the middle of the column and minimum velocity near the wall. This minimum local point provides a recirculation area in the column, which provides better mixing of the liquid. The CFD method can specifically calculate all local points with information on the recirculation area. For better optimization and understanding of the local point, AI can provide better information after training, and this method can map all input and output parameters.

Increasing the resolution, the more data of the fluid can be found. This needs more mesh density, smaller time steps, and consequently more computational effort in the CFD method. However, this is not the case by using the ANFIS method.

## Conclusions

The multiphase flow in a three-dimensional bubble column reactor (BCR) was studied using a three-dimensional CFD model and the ANFIS method. The Euler–Euler CFD model was used to simulate the flow pattern and general behavior in a cylindrical three-dimensional bubble column reactor. The CFD simulation results were used in the ANFIS training process. In the ANFIS method, the x, y, and z coordinates of the locations of the nodes were selected as inputs, and the liquid velocity was selected as the output variables. Inputs were evaluated in three modes, including one-dimensional (one input), two-dimensional (two inputs), and three-dimensional (three inputs). Using one-dimensional and two-dimensional inputs, the ANFIS method did not show the proper predictive capability. However, with three-dimensional inputs, the ANFIS method showed a perfect prediction. It also concludes that computational time can be reduced again when using three-dimensional inputs because the technique can be less intelligent with fewer data and rules and fewer iterations. The test data show an excellent agreement between the test and the ANFIS outcomes. This indicates the ability of the ANFIS method to predict the amount of fluid speed inside the reactor. Hence, the ANFIS technique shows that computational time effort can be significantly reduced, and this can significantly help speed up design, scale-up, and optimization. The ANFIS technique can also simulate hydrodynamics with the cheap computational cost.

Flow characteristics in the domain can be visualized by AI framework. However, the correct selection of AI parameters, as well as a suitable number of input parameters and data set can significantly change the computational time. The main limitation of the AI method is near the non-resolved part of the domain. In other words, new phenomena cannot particularly capture with AI and this method can only optimize the process. Besides the AI method, more online recording data through mechanical or chemical devices enables us to modify the prediction process. The AI calculation and analysis show with a greater number of input parameters the accuracy and stability of method rise significantly. As the AI method needs to get enough accuracy for prediction, small number of input parameters and data set can significantly increase computational time. Additionally, by increasing the number of inputs in AI framework, the stability of the method rises in terms of accuracy.

## Abbreviations

${C}_{D}$: Coefficient of drag force for dispersed phase [–]
${C}_{TD}$: Turbulent dispersion coefficient for dispersed phase [–]
${C}_{\varepsilon 1}$: Turbulent dissipation energy equation [–]
${C}_{\varepsilon 2}$: Turbulent dissipation energy equation [–]
${C}_{\mu }$: Constant in turbulence modelling of dispersed phase [–]
${C}_{\mu ,BI}$: Constant in bubble induced turbulence modelling of dispersed phase [–]
${d}_{B}$: Dispersed phase size [m]
$D$: Size of reactor [m]
D_S_: Ring sparger size [m]
$g$: Gravitational force in modelling [m s^–2^]
$H$: Height of reactor in modelling [m]
$k$: Turbulent kinetic energy [m^2^ s^–2^]
$${M}_{I}$$: Interfacial force [N m^–3^]
${M}_{D}$: Drag force for modelling of dispersed phase [N m^–3^]
$P$: Pressure in the reactor [N m^–2^]
$$U$$: Fluid velocity [m s^–1^]
$$w$$: Weight fraction [–]
$$CC$$: Correlation coefficient [–]
MF: Membership function for ANFIS
RMSE: Root mean square error for ANFIS

## Greek symbols

$\varepsilon$: Turbulent energy dissipation rate per unit mass [m^2^ s^–3^]
$\in$: Phase hold-up (–) [–]
$\stackrel{-}{\in }$: Average phase hold-up (–) [–]
$\mu$: Molecular viscosity [Pa s^–1^]
${\mu }_{BI}$: Bubble induced viscosity [Pa s^–1^]
${\mu }_{eff}$: Effective viscosity [Pa s^–1^]
$\rho$: Density of phases [kg m^–3^]
${\mu }_{T}$: Turbulent viscosity [Pa s^–1^]
${\tau }_{k}$: Shear stress of phase k [Pa]
${\epsilon }_{g}$: Volume of dispersed phase [–]

## Subscripts

$$G$$: Dispersed phase
$$L$$: Continuous phase
